# Advances in Dry Eye Disease Examination Techniques

**DOI:** 10.3389/fmed.2021.826530

**Published:** 2022-01-25

**Authors:** Yaying Wu, Chunyang Wang, Xin Wang, Yujie Mou, Kelan Yuan, Xiaodan Huang, Xiuming Jin

**Affiliations:** Eye Center, School of Medicine, 2nd Affiliated Hospital, Zhejiang University, Hangzhou, China

**Keywords:** dry eye, examination, tear, meibomian gland, demodex mites

## Abstract

Dry eye-related ocular surface examination is very important in the diagnosis and treatment of dry eye disease. With the recent advances in science and technology, dry eye examination techniques have progressed rapidly, which has greatly improved dry eye diagnoses and treatment. However, clinically, confusion remains about which examination to choose, how to ensure the repeatability of the examination, and how to accurately interpret the examination results. In this review, we systematically evaluate previous examinations of dry eye, analyze the latest views and research hotspots, and provide a reference for the diagnosis and management of dry eye.

## Introduction

Dry eye is a multifactor disease defined as “the loss of tear film homeostasis accompanied by ocular symptoms, caused by tear film instability and hyperosmolar, ocular surface inflammation and damage, and neuroparesthesia” (1). The global prevalence of dry eye disease is 5–50% depending on the population and disease definition, whereas in the United States, over 16 million adults have been diagnosed with the disease (2). Traditionally, dry eye has been classified as aqueous deficiency, evaporative, or mixed mode. This classification has been extended to take into account anatomical contributions to signs and symptoms, including neurological abnormalities (1). Common diagnostic methods include consideration of the chief complaint, a questionnaire, a physical examination, a tear film rupture test, corneal punctate staining, and a tear secretion test (3–5). Others include tear osmotic pressure and the tear ferning test (6–8). However, to an extent, the pathogenic factors and disease development of dry eye are complicated, and the separation of subjective feeling and objective signs is often encountered in clinical practice, which brings certain challenges to the diagnosis and treatment of the condition (9–14). With the advent of the technological era and prolific screen reading, the incidence of dry eye has increased (15, 16). As a result, greater attention is being paid to dry eye, so clinics have in turn developed more sophisticated means of checking for the condition through more targeted point-of-care tests and imaging technologies to help clinical doctors diagnose the type and severity of dry eye more effectively (17). In view of the importance of the systematic examination and evaluation of dry eye, in this paper, we summarize the relevant examination methods currently known, evaluate the new technologies available, and explore the progress of dry eye diagnostic methods, which may be helpful in the clinical diagnosis and treatment of dry eye.

A worker must first sharpen their tools if they want to do well. The examination of dry eye is very important for the diagnosis and treatment of dry eye. With recent advances in science and technology, the examination of dry eye has progressed rapidly, which has greatly improved dry eye diagnosis and treatment. In this paper, we summarize and analyze the latest progress in dry eye examination, which may be helpful in the clinical diagnosis and treatment of dry eye.

## Tear Examination

The normal tear film is made up of three layers: the lipid layer, the aqueous layer, and the mucin layer. Tears play an important role in maintaining the stability of the ocular surface microenvironment. Changes in the quality or quantity of tears will lead to the occurrence of dry eyes, and tear supplementation is an important part of dry eye treatment. The examination of the quality and quantity of tears is therefore an important indicator for the diagnosis of dry eye. In addition to the traditional tear secretion test and tear river height [tear meniscus height (TMH)] measurement, tear osmotic pressure, tear inflammatory factors, and the tear ferning test play important roles in the diagnosis of dry eye (18).

### Tear Examination

#### Schirmer's Test

Schirmer's test has been used as a diagnostic test for dry eye disease since 1903 and can help to evaluate tear volumes (19). The test is based on the physical tendency of a fluid to travel along a strip of porous material by capillary action due to surface tension (20–22). No consensus exists on the diagnostic criteria of dry eye in Schirmer's test. A reading of <5 mm is considered to indicate dry eyes and <10 mm marginally dry eyes. Schirmer's test measures total tear secretion, including reflex, and basal tears. The test is known to measure reflex tears without anesthesia and basal tears with anesthesia (23–25). However, the concept of basal tears is uncertain. It may not be necessary to measure true basal tears with anesthesia because even light stimulation can change tear secretion (26–28).

Schirmer's test is popular for diagnosing dry eye because of its simple operation and the absence of a requirement for equipment. However, the disadvantages include poor repeatability, low sensitivity and specificity, and sharp patient discomfort (29). The change in light, room humidity, and temperature and patient's anxiety may explain the poor repeatability. Furthermore, the variability in Schirmer's test results may be caused by reflex tearing (23). To increase the reliability of Schirmer's test, many variations in Schirmer's test have been proposed. In a comparative study of Schirmer's test with and without anesthesia, Kashkouli et al. found that the value of Schirmer's test with anesthesia may be more objective and reliable than that without anesthesia (30), and they discovered that a shorter 1-min test with anesthesia not only decreased patient's discomfort but also saved time. Hamano et al. modified Schirmer's test using a cotton thread impregnated with phenol red dye instead of uncomfortable filter paper (31). In this test, which is called the phenol red thread (PRT) test, the color of the dye changes from yellow to red depending on the pH of the tears (21). However, the relationship between the PRT and Schirmer's test in the measurement of tear secretion is not clear (27). Although Schirmer's test without anesthesia may not be acceptable given its shortcomings, it can be considered a valid option for diagnosing severe dry eye because of its reproducibility (32–34).

In view of the shortcomings of the tear secretion test, nonimmersive and improved tear detection, tear composition, physical and chemical properties, and other aspects of inspection and evaluation may be new directions for exploration.

#### Tear Meniscus Volume

The tear menisci provide a reservoir that contributes to the formation of the preocular tear film with each blink and accommodates excess tears during reflex tearing, lacrimal obstruction, and after topical drop instillation (35). It has been reported that the tear meniscus contains 75–90% of the aqueous tear volume, which is positively correlated with the lacrimal secretory rate (36). The meniscus volume is also reported to be reduced in tear-deficient dry eye (37, 38). Thus, the quantitative assessment of tear meniscus parameters may be useful in the diagnosis of dry eye disease. Tear meniscus variables, such as height, width, cross-sectional area, and meniscus curvature, have been reported to be of value in the diagnosis of dry eye (39, 40). TMH and strip meniscometry (SM) can be used to evaluate tear meniscus volume.

##### Tear Meniscus Height

**Figure 1:**
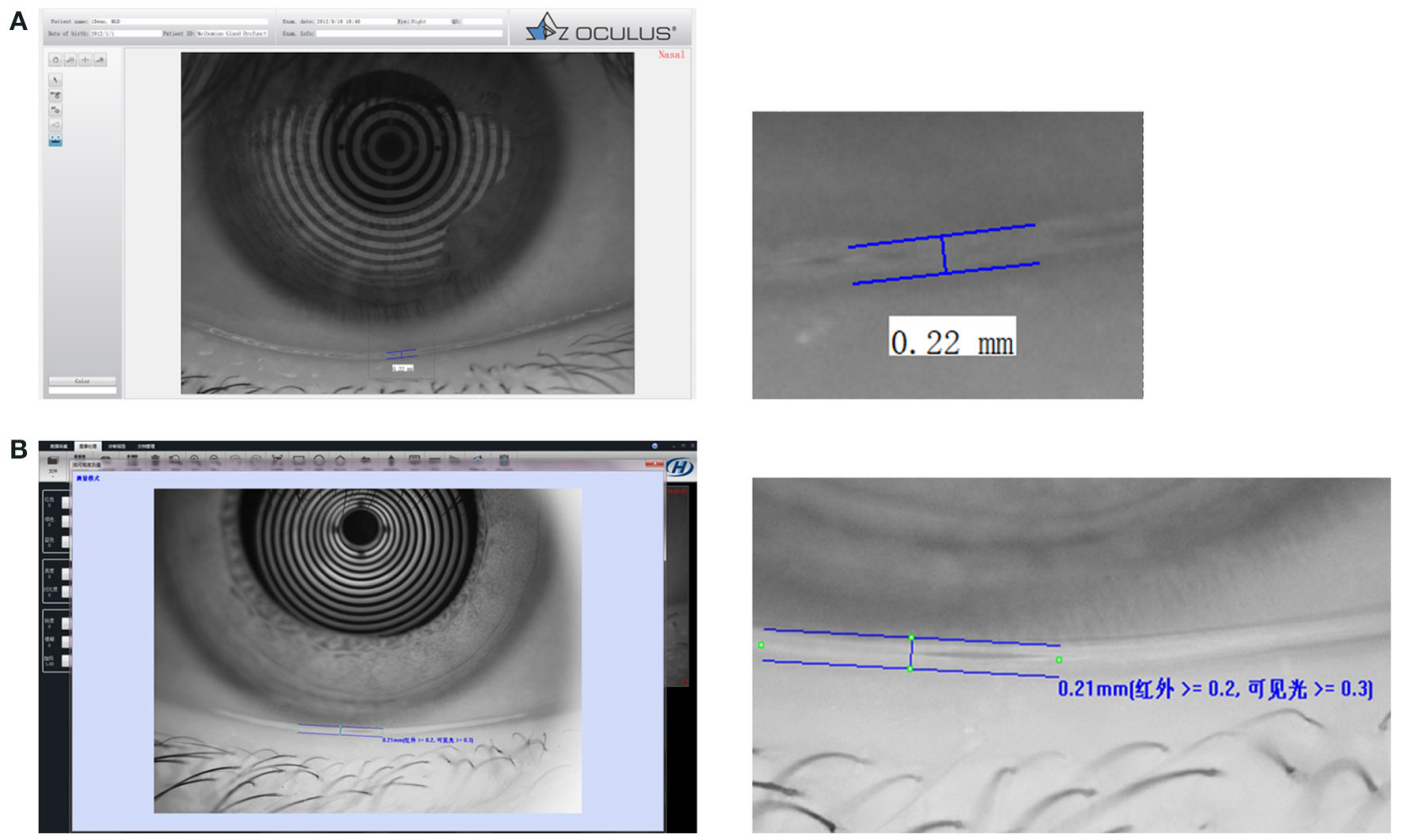
Tear film thickness. **(A)** An example of tear film thickness from OCULUS Keratograph 5M. **(B)** An example of tear film thickness from Kanghua dry eye analyzer.

Tear meniscus height has been confirmed to have good reliability and accuracy in detecting tear meniscus volume (41, 42). Invasive and noninvasive techniques can be used to measure TMH (see Figure 1). TMH can be viewed under cobalt blue light using a slit lamp and fluorescein instillation (43, 44). However, invasive TMH assessments are challenging as a result of their limitations. Frequent blinking, drug stimulation, time intervals, humidity, and temperature have been reported to influence invasive TMH results (45–47). Noninvasive TMH was therefore introduced, and optical coherence tomography (OCT) measurement of the tear menisci was found to be more reliable and was validated. Noninvasive TMH can be used together with noninvasive tear breakup time (NIBUT) on the same instrument, and they have been shown to be positively correlated (48, 49).

##### Strip Meniscometry

**Figure 2:**
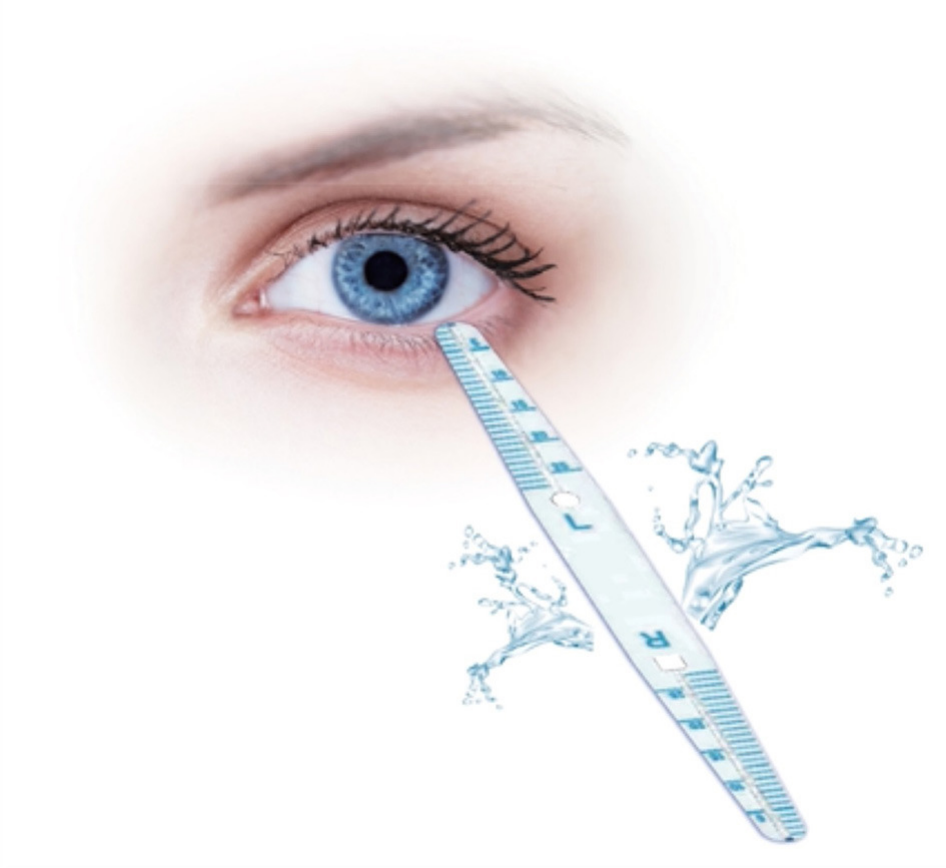
SMTube. The method of strip meniscometry (SM) is using SMTube which is a thin strip (length: 85 mm, width: 7 mm, and height: 0.3 mm) with a capillary absorber in the center and two columns of scale on both sides to measure the tear meniscus volume. The examiner held the center part of the strip and immersed the tip into the tear meniscus of the lower eyelid for 5 s to absorb tears. The SMTube attached to the tear meniscus absorbed the tears into the ditch and the strip color turned blue, indicating the volume. At the end of 5 s, the strip was taken out and the blue stained column length was measured. The length of the stained column for normal people was equal to or >5 mm, whereas it was <5 mm for DED patients.

Strip meniscometry (39, 40, 50–52) involves using an SMTube, which is a thin strip (length: 85 mm, width: 7 mm, and height: 0.3 mm) with a capillary absorber in the center and two columns of scale on both sides, to measure tear meniscus volume. The examiner holds the center part of the strip and immerses the tip into the tear meniscus of the lower eyelid for 5 s to absorb tears. The SMTube attached to the tear meniscus absorbs the tears into the ditch, and the strip color turns blue, indicating the volume. At the end of 5 s, the strip is taken out, and the blue-stained column length is measured. The length of the stained column for normal people is equal to or >5 mm, whereas it is <5 mm for patients with dry eye (see Figure 2). Dogru et al. (39) and Miyasaka et al. (53) demonstrated that SM results correlate with those of Schirmer's test. A good correlation between TBUT and SMTube measurements has also been reported (52). The SMTube is useful for the diagnosis of dry eye, and its validity can be evaluated effectively using the CASIA SS-1000 AS-OCT TM parameters. Lee et al. (50) compared tear meniscus measurements using SM and the Keratograph 5M between 3 patient groups with subtypes of dry eye disease and found that tear meniscus measurements using SM and the Keratograph 5M can compensate for the detection of aqueous-deficient components of dry eye (53). The SMTube is useful for evaluating tear volume and therapeutic effects in patients with lacrimal passage obstruction. Hao et al. (40) showed that the SMTube has acceptable repeatability and reproducibility and specific correlations with TMH, BUT, and Schirmer's test. SM can provide clinical staff in busy outpatient services with a swift and convenient inspection method.

#### Tear Ferning Test

**Figure 3:**
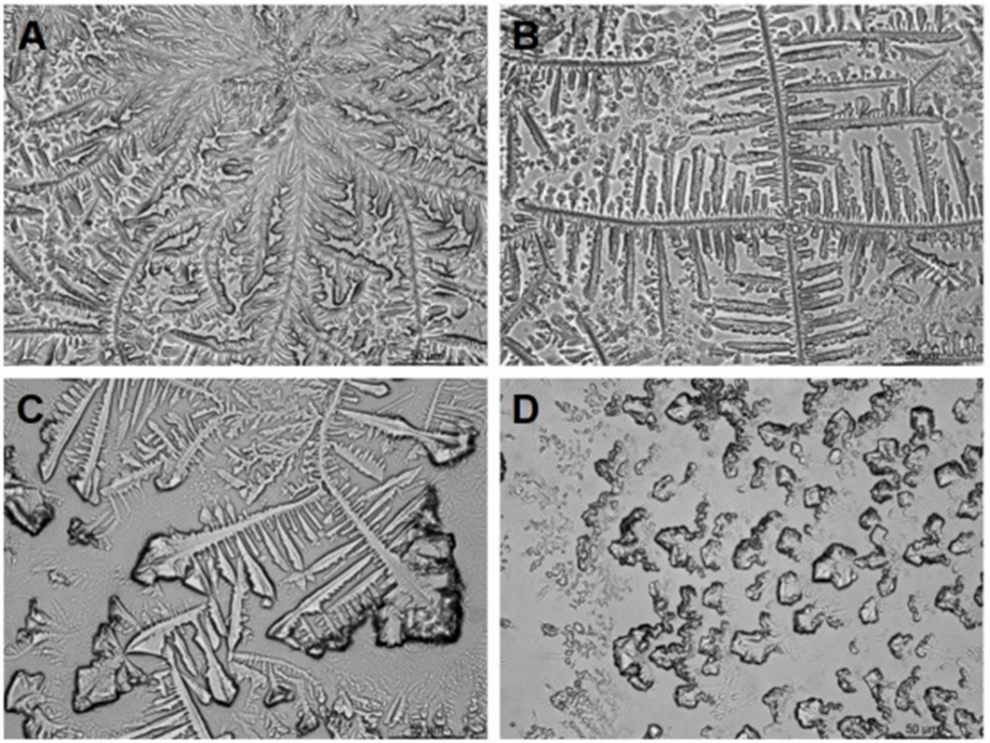
Tear ferning test classification descriptions (Rolando's classification). Type 1: uniform arborization in the entire field of observation without spaces between the ferns. Single ferns are big and closely branched **(A)**. Type 2: Arborization is abundant, but the single ferns are smaller and have a lower frequency of branching than in grade 1; empty spaces appear between the ferns **(B)**. Type 3: Single ferns are little and incompletely formed with rare or no branching **(C)**. Type 4: No ferning is present; mucus may appear in clusters and threads **(D)**.

The tear ferning test is a simple tear detection method that can reflect some biochemical characteristics of tear film relatively quickly and cheaply (see Figure 3) (44, 54). Golding et al. (55) pointed out that the salts and polymers in tears are the important factors affecting the crystallization of tear ferns in the microscopic photographic study of tear crystal patterns in the tear ferning test. In their analysis of normal tears, they found that the fern-like crystals are mainly composed of sodium chloride, potassium chloride, and trace ions, and the surface of the crystals is covered by mucins and high molecular proteins, which indirectly control the formation of the crystals. Kogbe et al. (56) found that fern-like crystal patterns and branching patterns are particularly dependent on the ratios of monovalent sodium and iron ions to divalent calcium and magnesium ions. The tear fern crystal map in the tear ferning test is a relatively inexpensive and quick test of the total biochemical level of tear samples. Traipe-Castro et al. (57) studied the dynamics of tear evaporation and found that the crystalline pattern of dry healthy tears can be divided into four structural regions, namely zone 1 (outermost), a transition zone, zone 2 (near central region crystal), and zone 3 (central main crystal). The crystal pattern of ferns produced by a single tear sample depends not only on the composition of the tear, but also on the environmental conditions of the dry tear. Reducing the pressure and humidity or increasing the temperature to speed up the drying speed of the tear results in a smaller crystal morphology and different proportions of the four structural regions in the crystal pattern. In addition to diagnosing dry eye, the tear ferning test can be used to diagnose keratoconjunctivitis and cystic fibrosis disease and for soft contact lens tolerance prediction in clinical practice (58–60). The tear ferning test has good clinical guiding significance. However, the current method has disadvantages, such as the difficulty in controlling environmental conditions and the slow speed of the experimental results. If a method for rapid crystallization could be found, this examination method may have more clinical value.

#### Tear Osmotic Pressure

The measurement of tear osmotic pressure indicates the balance among tear secretion, evaporation, absorption, and drainage (61). Vapor pressure osmometry and freezing point depression have been used to measure tear osmotic pressure in the past (62, 63). The tear osmolarity test is considered as one of the most accurate dry eye diagnosis methods (64). Most recent techniques to measure tear osmotic pressure involve the use of the TearLab (TearLab Corporation, Escondido, CA) and I-PEN (I-MED Pharma Inc., Dollard-des-Ormeaux, Canada) osmolarity systems (65, 66).

##### TearLab Osmometer

The TearLab osmometer (TearLab, San Diego, CA) was approved by the US Food and Drug Administration in 2008 for the measurement of tear osmolarity *in vivo* by clinical practitioners. Since then, several studies have demonstrated that it is a reliable test with good performance and complements for the diagnosis of dry eye (67–69). Some studies have concluded that the TearLab osmometer is the best single marker for diagnosing and classifying dry eye levels of severity and for distinguishing between different dry eye severity levels (70, 71). On the other hand, several clinical studies have raised questions about the diagnostic ability of tear osmolarity in dry eyes measured with the TearLab osmometer (72–77).

##### I-PEN Tear Osmolarity Test

Tear osmolarity is performed using the I-PEN osmolarity system 5 min after a PRT test. The I-PEN osmolarity system is used a distance away from electronic devices to ensure the accuracy of the readings. Each subject is asked to close their eyelids gently for 30 s, and the disposable single-use sensor then softly makes contact with the palpebral conjunctiva from the lower eyelid at a 30° angle. By design, the I-PEN beeps after a few seconds and displays an osmolarity reading on the screen (78, 79). Tear osmolarity is measured three times in the right eye of each subject with 5-min intervals between measurements. Based on the I-PEN tear osmolarity measurements, subjects are classified as having a healthy eye (<290 mOsm/L), minor dry eye (290–310 mOsm/L), mild dry eye (310–330 mOsm/L), and moderate dry eye (330–350 mOsm/L).

Shimazaki et al. (78) studied the efficacy and safety of the handheld I-PEN in Japanese patients with dry eye disease and those without dry eye disease and found no correlations between the tear film osmolarity values obtained with the I-PEN system and any subjective or objective parameters of dry eye. Meanwhile, in the study of Fagehi et al. (79), the mean measurement of I-PEN tear osmolarity was 303.8 ± 4.8 mOsm/L, which is in agreement with the range reported for healthy subjects. The I-PEN is reliable and has the advantage of portability compared to other osmolarity systems (79). Park et al. (80) obtained similar results, indicating that the I-PEN osmometer can be considered suitable for the use in clinical settings, with good performance in the diagnosis of dry eye. König et al. (81) found that the I-PEN osmometer provided significantly higher tear film osmolarities than those measured using the TearLab osmometer. In one study, tear osmotic pressure measured using the I-PEN osmometer has shown a significant decrease after treatment with a botulinum toxin A injection in patients with intractable dry eye disease (82). Tavakoli et al. found that, *in vivo*, both instruments displayed poor repeatability (83). Some studies have found that after treatment, the osmotic pressure of tears in patients with dry eye is significantly reduced (74–86). However, other studies have found that the osmotic pressure of tears does not change significantly after treatment (84–88). This may be related to the duration of the treatment, the severity of the disease, and the efficacy of the different treatments. The key elements in the diagnosis of dry eye are increased osmolarity of the tear film and inflammation of the ocular surface, which are accompanied by ocular symptoms. In terms of clinical treatment, we can select different artificial tears based on the osmotic pressure of patients with dry eye.

#### Tear Inflammatory Factor

There is very strong and valid evidence that inflammation constitutes an important pillar in the pathophysiology of dry eye. Increased inflammatory cells, increased expression of immune activation and adhesion molecules, T-helper type 1 (Th-1) and Th-17 attracting immune pathways, cytokines, and chemokines are all evidence supporting the inflammatory pathology of dry eye (89, 90).

Currently, the only commercial options to investigate tear film biomarkers are InflammaDry® (matrix metalloproteinase 9, MMP-9) and the TearScan™ Lactoferrin Diagnostic Test Kit (91). MMP-9 is an endopeptidase that helps remodel the extracellular matrix and plays a crucial role in dry eye. The point-of-care test InflammaDry (Quidel, San Diego, CA) can detect this biomarker in tear film with a low limit of detection of 40 ng/mL (91). This test should be carried out prior to the use of anesthetics, corneal staining, or Schirmer's test. A fleece placed at the tip of a sample collector is inserted multiple times along the patient's palpebral conjunctiva. The sample collector is then placed into the supplied test cassette and secured prior to immersing it in the buffer. Once 10 min has passed, the test cassette may display a blue line, which represents a valid test. If the blue line does not appear, the test is considered as invalid and must be repeated. A positive result is indicated by the presence of pink and blue lines, thus providing a qualitative (yes or no) response. To carry out this test accurately, a sufficient sample (5 μL) needs to be collected to avoid a false-negative result. In some studies, the InflammaDry test has demonstrated high positive and negative agreement for confirming suspected dry eye disease (92, 93). These studies have further shown that tear MMP-9 positivity may serve as a reliable response predictor of topical corticosteroid treatment in dry eye. Kim et al. (94) found that the subjective 5-scale grading system in the point-of-care MMP-9 immunoassay is an easy and reliable method with acceptable accuracy. Whereas, inflammation is known to play a role in dry eye, it is not always present in those with symptoms. Lanza et al. (95) suggested that a clinical examination alone cannot identify patients with clinically significant inflammation. In their study, there was no difference in the dry eye profiles of patients when evaluating symptoms and signs and those who tested positive vs. negative for MMP-9 on the ocular surface. A randomized, double-blind, placebo-controlled dry eye study in humans reported that lactoferrin supplementation can increase tearing, whereas a randomized controlled study of cataract surgery-induced dry eye found that it can restore tearing and TBUT (96, 97).

### Tear Film Examination

The most superficial layer of tear film is the lipid layer secreted by the meibomian glands. When this layer is deficient, it may result in an evaporative dry eye (18). The main methods of tear film examination are TBUT and tear film thickness, but the correlation between these two examinations needs further investigation.

#### Tear Breakup Time

Tear breakup time has been the most frequently adopted dry eye examination method over the past two decades (98). TBUT was first introduced by Norn (99) to assess the stability of tear film. It is traditionally defined as “the interval between the last complete blink and the first appearance of a dry spot or disruption in the tear film” (99). According to current perspectives, measurements of TBUT can be divided into two distinct patterns: invasive and noninvasive.

##### Invasive Tear Breakup Time

The most common clinical method of measuring invasive TBUT is sodium fluorescein (100). The general process involved in examining TBUT with fluorescein is convenient and accessible. Liquid containing light fluorescence or a sterile fluorescein paper strip infiltrated with normal saline is presented to the superior bulbar conjunctiva for 1–2 s so that the fluorescein is uniformly distributed on the precorneal ocular surface (101, 102). This study has shown that the volume of fluorescent liquid used can significantly affect TBUT results, so a micropipette can be used to control the volume (102). The use of a dry fluorescein applicator is also an effective way to minimize the influence of liquid volume (46, 103). After the patient blinks several times, the time from the last blink of the eye to the first dry spot on the tear film is measured under a cobalt blue filter. Two or three consecutive measurements are recorded. The average value provides a more reliable result (104). A recent study showed that the maximum TBUT may also be considered to diagnose dry eye (105). The sensitivity and specificity of the TBUT test are 72 and 61%, respectively. The diagnostic criteria for fluorescein TBUT in diagnosing dry eye are <10 s (105). Generally, fluorescein TBUT values can be expected to be shorter than the values of noninvasive TBUT. A fluorescein TBUT of <5 s has also been reported to contribute to the diagnosis of dry eye (106).

##### Noninvasive Tear Breakup Time

In recent years, due to the weak points of invasive TBUT, NIBUT, which relies on technical apparatus, has become more popular (107). Invasive TBUT may be affected by volume, the concentration and types of fluorescence used, and the experience of the examiner (108, 109). NIBUT does not involve the instillation of any substances nor is there any direct physical contact with the tear film and conjunctiva. NIBUT is measured by the automated capture of Placido disk images, which are obtained from the anterior ocular surface using a corneal topography system (110–113). Computer software is used to identify irregularities and the breakup of the precorneal tear film, and the first and average time is calculated (114, 115). These several studies have reported better sensitivity and specificity for NIBUT than invasive TBUT. NIBUT has 82–84% sensitivity and 76–94% specificity (116). In addition, 10 s has been proposed as the cutoff value, and <10 s can indicate a diagnosis of dry eye (117). The disadvantage of NIBUT using technical apparatus is that, instead of repeating the test three times, a random time is checked once, so there is room for error, and this method therefore needs to be improved in the future.

#### Tear Film Thickness

A stable eye tear film is a sign of good eye health, mainly because it forms the main refractor through which light enters the visual system and protects and moistens the cornea. Wolff (118) proposed a three-layer model of tear film: the mucin layer covering the eye surface reduces the hydrophobicity of epithelial cells; care of the water layer of exposed ocular epithelium by providing lubricity, some nutrients, antimicrobial proteins, and appropriate osmotic pressure; Additionally, the lipid layer prevents the water layer from being lost through overfilling and evaporation. When the eyes are open, the tears are distributed in three compartments, which are the fornical compartment, the tear menisci, and the preocular tear film (118). The preocular tear film overlies the exposed conjunctiva and cornea (119, 120).

**Figure 4:**
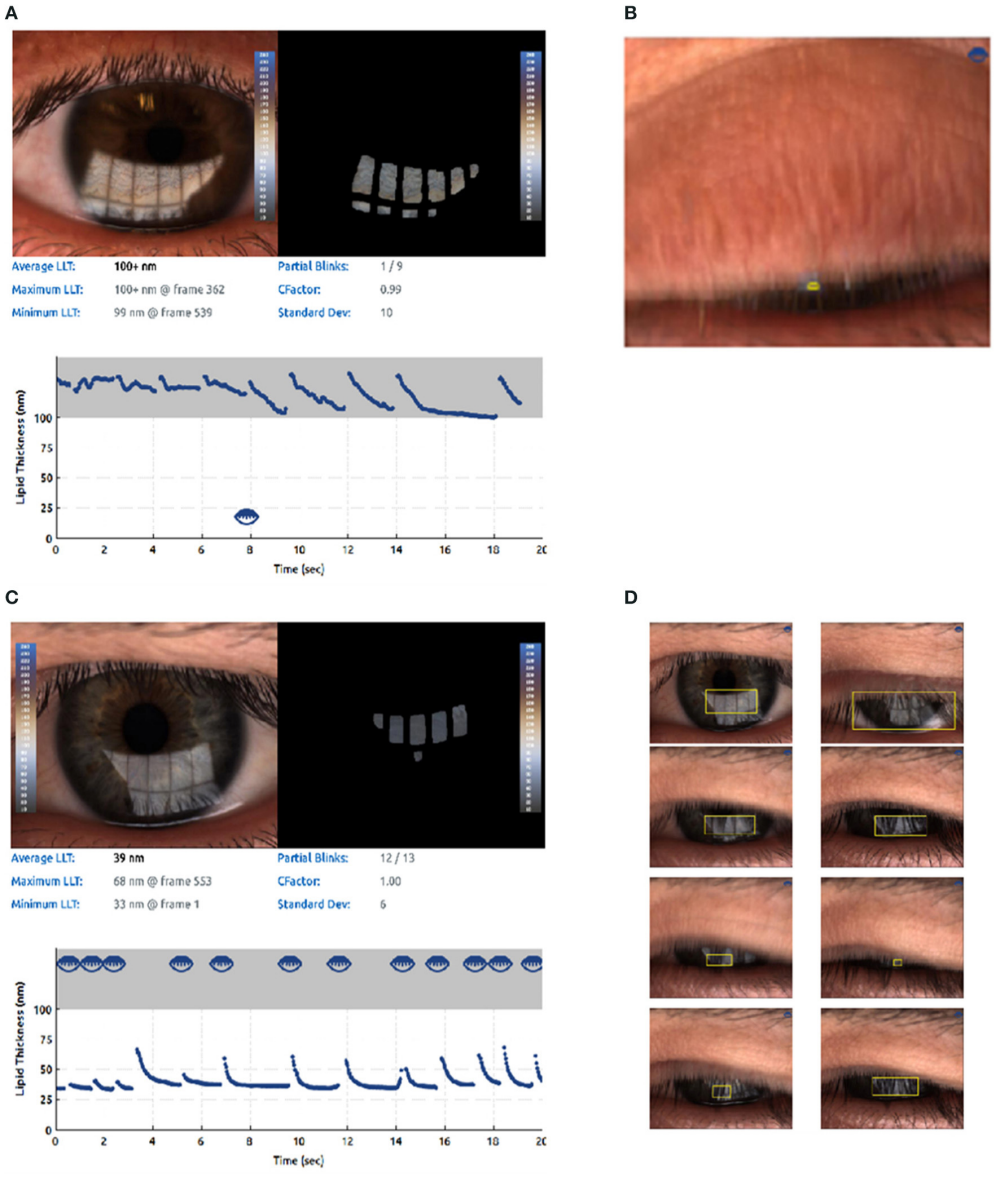
Lipid imagine report and incomplete blinking (an example from LipiView II ocular surface interferometer). The computer system captures a video image file that is recorded over time since the interference pattern changes as the tear film is distributed across the cornea during blinking. **(A)** Lipid imagine report and incomplete blinking. **(B)** Partial blink imagines. **(C)** Another for lipid imagine report and incomplete blinking. **(D)** Another for partial blink imagines.

Measurement of the lipid layer thickness (LLT) could serve as an useful examination method in clinical practice diagnosing MGD. In previous clinical routine, the LLT is usually measured indirectly by the determination of the TBUT. Nowadays, various technologies and new devices are currently being utilized to assess LLT. It is 2–5.5 μm thick in the corneal region. A suitable method for the direct quantification of the LLT is interferometry techniques (120–122). LipiView II interferometer (TearScience Inc, Morrisville, NC) could provide the clinician with the important output parameter, such as average LLT of the tear film and be capable of quantifying LLT (123). On the other hand, LipiView II could automatically detect and analyze blink rate and blinking quality through the videos recorded. It displays the number of complete blinks and incomplete blinks and blink frequency numerically to help clinician analyze blinking pattern and take images of MG for visualizing the morphology of the MGs (see Figure 4). Compelling visuals and video captures provide an opportunity to educate patients about their personal ocular health and how healthy meibomian gland function protects the ocular surface, keeping their eyes moist to ensure clear, comfortable, healthy, and stable vision (124). Willcox MDP et al. pointed that abnormal blinking habits such as partial blinking are strongly associated with MGD (125). Using meibography, the grading of dropout at baseline and subsequent examinations can be used to track long-term progression of MGD (62). A study compared the Subjective Keeler Tearscope-Plus™ with the Objective Oculus^®^ Keratograph 5M and LipiView^®^ and found that the results of tear stability or lipid thickness were interchangeable (126). Another study compared the LipiView ^®^ II with IDRA^®^ for ocular surface analysis and found no significant difference in LLT, but these devices should not be used interchangeably for the evaluation of meibomian gland dropout and partial blink rates (127).

### Examination of the Eyelid Margin and Meibomian Glands

Meibomian gland dysfunction (MGD) is the main cause of dry eye, with 86% of dry eye cases being caused by MGD. Meibomian gland dropout is significantly correlated with the other clinical features of MGD, such as the quality of the expressed meibum, altered tear film lipid layer stability, and ocular surface damage. Meibomian gland morphology and function are routinely examined to diagnose MGD (128). Using meibography, the grading of the dropout at baseline and subsequent examinations can be used to track the long-term progression of MGD (129–131). Evaluating changes in the eyelids, eyelid margins, and meibomian glands is of great value in the diagnosis of MGD. These can be assessed by observing the opening state of the meibomian glands, squeezing the meibomian glands on the eyelid skin, and observing the difficulties and character of eyelid ester excretion. Specific scoring criteria were established following the consensus of experts on the diagnosis and treatment of MGD in China in 2017 (129).

#### Eyelid Margin Examination

Patients with MGD are prone to eyelid margin abnormalities. Abnormal eyelid margins can be valuable in diagnosing MGD. For example, MGD patients may have a thickened, blunt eyelid margin, an irregular shape, a Marx line (the junction of the skin and mucosa), a receded opening of the meibomian glands, hyperemia of the eyelid margin, and neovascularization. Slit lamp examination of the entire area of the upper and lower eyelids may reveal abnormal signs, including irregular eyelid margins (with notching along the eyelid margins), telangiectasis, and a shift of the mucocutaneous junction (130–132). Amano examined total MGD patients and noted that the slit lamp findings of lid margin abnormalities, a shift of the mucocutaneous junction, telangiectasia, and plugging were more frequent (133).

##### Meibomian Gland Infrared Meibography

**Figure 5:**
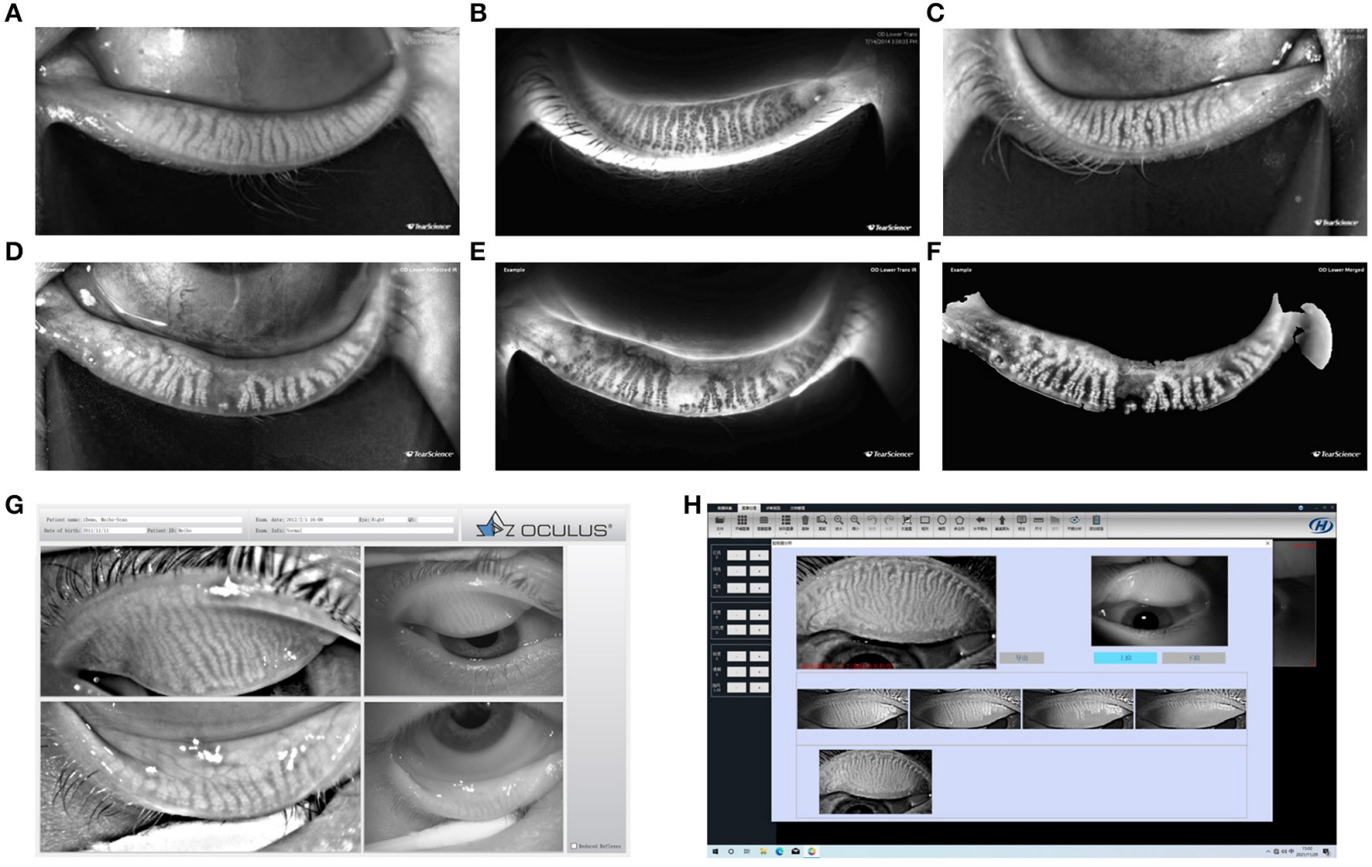
Meibomian gland morphology. Ocular surface interferometer—LipiView II interferometer **(A–C)**; Dynamic Meibomian Imager—LipiScan **(D–F)**; OCULUS Keratograph 5M **(G)**; Kanghua Dry Eye Analyzer **(H)**. Examples for LipiView II and LipiScan: The images of the glands may be viewed on the computer screen display and in a printed report. Three modes of gland images are provided: Reflected infrared view is shown for both upper and lower eyelid gland images. **(A,D)** Show only lower eyelid gland images. Transillumination infrared view is shown only for lower eyelid gland images, if images were captured using handheld near-infrared lid everter **(B,E)**. Merged view is the combination of the reflected infrared and trans infrared views and is shown only for the lower eyelid gland images **(C,F)**.

Several modalities of meibomian gland imaging are used in MG examinations, including contact meibography (not currently popular), noncontact infrared meibography (e.g., IR meibography and the mobile pen-shaped meibography system), keratography (Keratograph 5M, OCULUS, Wetzlar, Germany), Kanghua Dry Eye Analyzer (DED-1L, Kanghua, Chongqing, China) and LipiView II (TearScience, Morrisville, NC) (see Figure 5), *in vivo* confocal microscopy (IVCM, see Section Other Examinations), and OCT meibography (134). The MG area ratio, diameter deformation, tortuosity, and signal intensity could be considered as promising biomarkers for MGD diagnosis and objective grading (131). Meibomian gland infrared meibography may provide a reference for clinical treatment of MGD, especially nondrug treatment, such as Lipiflow, IPL, and meibomian gland massage.

#### Lid Wiper Examination

The lid wiper is a component of the conjunctival margin of the upper and lower eyelids (135). Shiraishi et al. noted that lissamine green staining was detected in more than 10% of lid wiper epitheliopathy patients with dry eye symptoms (136). Studies have suggested that the best staining method is as follows: lissamine green or fluorescein sodium test paper is soaked and used to make contact with the conjunctiva of the lower eyelid. After more than 1 min, the conjunctiva is restained once. The degree and range of staining of the lid margin epithelial cells are then examined under a slit lamp microscope with a cobalt blue light. A staining length of ≥2 mm and/or ≥25% of the eyelid margin width are considered positive in the diagnosis of lid wiper epitheliopathy (137).

To date, studies have found that the incidence of lid wiper epitheliopathy in patients with dry eye symptoms increases, and lid margin staining can be combined with traditional dry eye diagnosis methods to improve diagnosis rates.

#### Inspection of the Eyelid for Demodex Mites

Ocular mite infection is significantly correlated with dry eye, MGD, and age. When Demodex mites infect the margin of the eyelid, the skin of the margin of the eyelid, the hair follicles and glands of the eyelid, and the meibomian glands will accumulate mites, and symptoms related to dry eye will occur (138). In addition, a typical clinical manifestation of Demodex blepharitis is cylindrical dandruff at the root of the eyelash, and this is considered pathognomonic for Demodex blepharitis (139). To detect Demodex mites, an optical microscope is used to examine eyelashes with typical cylindrical dandruff characteristics. These lashes are then removed and placed on a slide to observe the number and morphology of the Demodex mites. The use of IVCM (see Section Other Examinations) could be an easy way to improve this diagnosis. The advantage of IVCM is that it can be used to quickly and simultaneously detect microorganisms and eyelid structure. It is noninvasive and does not require the removal of eyelashes. It can be used repeatedly to determine a prognosis in patients, but it cannot be used to accurately to identify the type of mite.

### Ocular Surface Staining

Ocular surface cell staining can evaluate the barrier function and integrity of epithelial cells as one of the evaluation indexes of the severity of dry eye (140). When the integrity of ocular surface cells has been damaged, they can be stained with specific dyeing agents to display the defects. The degree and area of staining are related to the severity of the ocular surface damage. Ocular surface cell staining can therefore be used to evaluate the barrier function and integrity of epithelial cells. Fluorescein sodium staining is commonly used in clinics although lissamine green staining and rose bengal staining are sometimes also used.

#### Fluorescein Staining

The mechanism of fluorescein staining involves dyeing corneal defects through the diffusion of the fluorescein between cells into the adjacent intercellular space with penetration into the lower stroma. Healthy corneal epithelial cells are therefore not stained unless they have defects. This method is mainly used to assist in the diagnosis of epithelial defect-related diseases, such as corneal abrasions, keratitis, and corneal erosion. It can also be used to evaluate the therapeutic effects of epithelial injury (140). Sodium fluorescein staining is not a specific index of dry eye but plays an auxiliary role in its diagnosis.

Sodium fluorescein is available in two forms: eye drops and impregnated paper strips. For both the eye drops and paper strip methods of instillation, the aim is to achieve highly fluorescent staining of the areas with loss of epithelial integrity (141). Studies have shown that high concentrations of sodium fluorescein are nonfluorescent and fluorescent at a concentration of <0.1% (142). It is therefore general practice for doctors to deliver 2 μL of 2% sodium fluorescein eye drops or fluorescein sodium strips soaked in physiological saline or other eye drops into the conjunctival sac using a micropipette. The whole tear film is then filled by the patient blinking their eyes. Ocular surface staining can then be observed using a blue exciter filter over the white light source and a complementary yellow or orange barrier filter over the slit lamp objective (142).

#### Rose Bengal Staining

A derivative of fluorescein, rose bengal is believed to not stain healthy epithelial cells. According to Roberts et al.'s study (143), the reason that the normal ocular surface does not stain with rose bengal is the obstruction of a healthy and intact ocular tear film. Accordingly, as long as the tear film protection is insufficient, there will be rose red staining. This feature can be used to assist in the diagnosis of dry eye. In a study by Korb (144), rose bengal was found to be the preferred dye for bulb conjunctival staining, whereas sodium fluorescein was the preferred dye for corneal staining, with an optimal concentration of 1%. Due to the cytotoxic effect of rose bengal, patients will feel tingling if anesthetic eye drops are not used. Furthermore, the sensitivity and specificity of rose bengal are not as good as those of sodium fluorescein, so it is not widely used and can only be used as a reference (137, 144, 145).

#### Lissamine Green Staining

Lissamine green staining has similar characteristics to rose bengal, but its cytotoxicity is much less than rose bengal, so lissamine green staining is commonly used as a substitute in conjunctiva staining in clinical practice (146, 147). These two dyes do not spread throughout the conjunctiva due to the blocking effect of the mucins in tear film, so their staining areas last longer than sodium fluorescein. Yoon found that a mixed solution of 1% fluorescein and 1% lissamine green can simultaneously stain the cornea and conjunctiva while maintaining the unique staining characteristics of each dye (147).

Notwithstanding, the use of mixed dyes may necessitate a long waiting time. The use of different dyes on the corneal and conjunctival epithelial cells may also have subtle effects on the damaged areas. Fluorescein sodium strips are therefore commonly used in clinical practice. Although reactive dyes have been used to stain the cornea and conjunctiva to assess ocular surface disease in dry eye for a long time, there is no uniform or clear ocular surface stain rating scale due to the different concentrations and methods of staining used in different regions. The general content is to classify the dyeing area, density, area, and type. Most studies use the Oxford scale, but the type of scale used depends on the researchers' requirements (148). Dyes are therefore an effective adjunct to the diagnosis of dry eye and to determine the type and severity of dry eye.

### Dry Eye Analyzer

**Figure 6:**
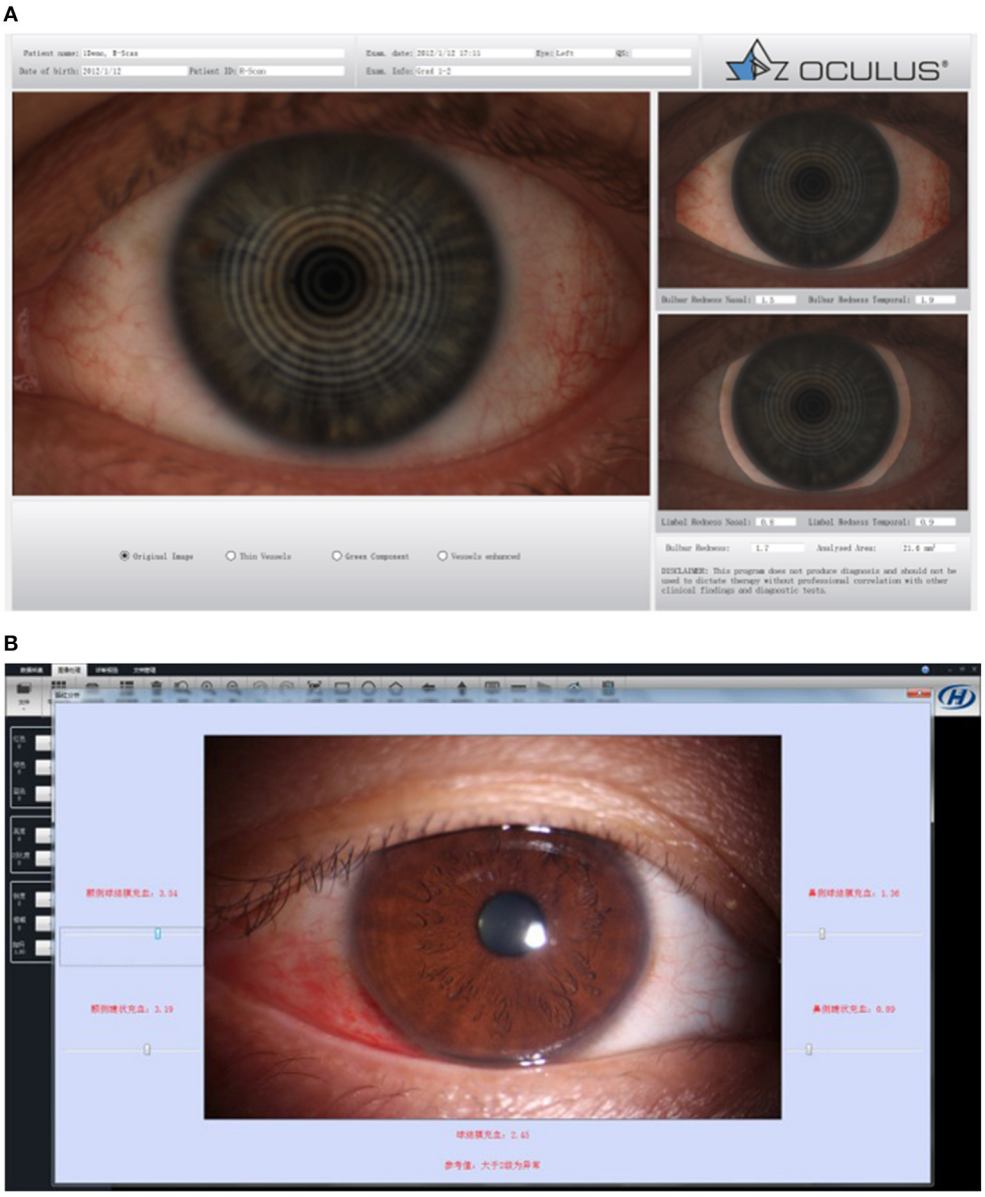
Bulbar redness. **(A)** OCULUS Keratograph 5M for bulbar redness grading scale. **(B)** Kanghua dry eye analyzer for bulbar redness grading scale.

**Figure 7:**
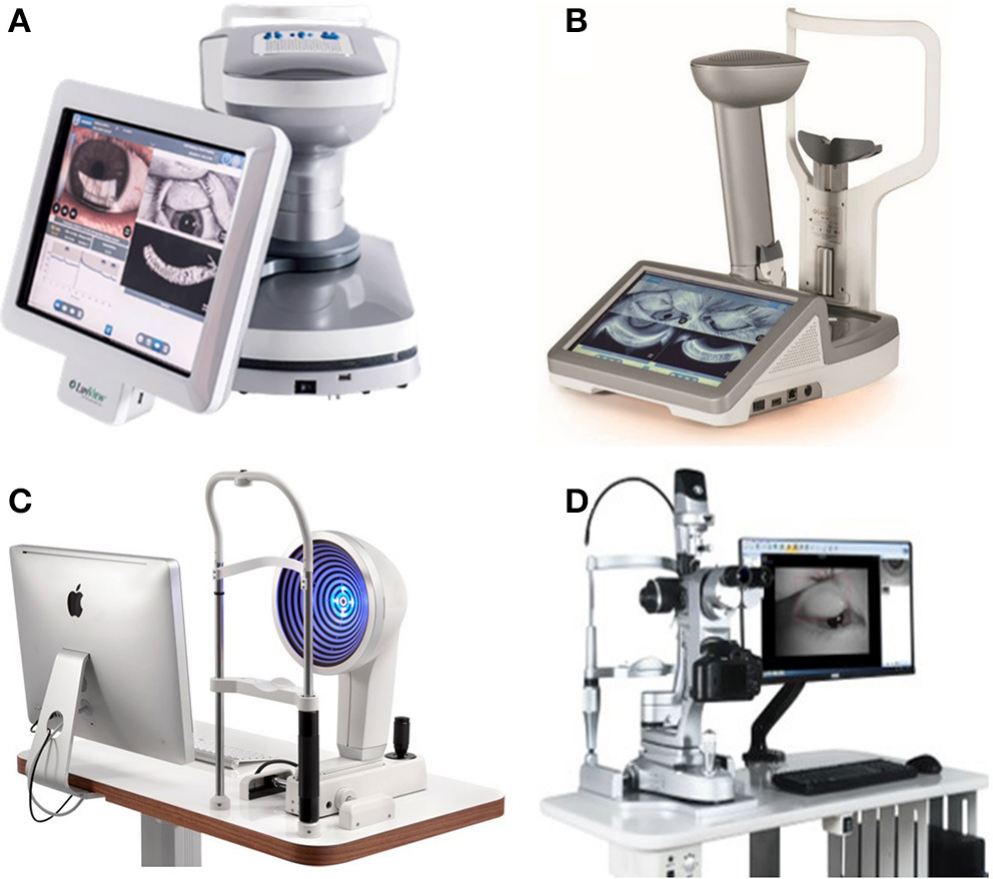
Dry eye analyzers. **(A)** LipiView II ocular surface interferometer. **(B)** LipiScan dynamic meibomian imager. **(C)** OCULUS Keratograph 5M. **(D)** Kanghua dry eye analyzer.

The dry eye analyzer is a device that integrates a variety of dry eye examinations. The dry eye analyzer can provide fast, convenient, and efficient dry eye examinations, but its relationship with traditional examinations needs to be clarified. Dry eye analyzer examinations may include tear river height (TMH) (see Section Tear Breakup Time), NIBUT (see Section Tear Breakup Time), tear film thickness (see Section Tear Film Thickness), infrared photography of the meibomian glands (see Section Eyelid Margin Examination), and conjunctival hyperemia analysis (see Figures 6, 7).

#### Conjunctival Hyperemia Analysis

Bulbar hyperemia is a common clinical sign and important indicator of ocular disease (149). It is one of the most common contributors of ocular complaints that prompt visits to medical centers. The translucent appearance of the conjunctiva allows immediate observation of changes in microvascular circulation. Conjunctival congestion is caused by dilation of blood vessels in microangiopathic degeneration caused by an inflammatory response. Our understanding of these neurogenic and immune-mediated pathways has progressed over time, and they are now known to play a critical role in the development of targeted novel therapies. Due to a multitude of underlying etiologies, patients must be accurately diagnosed for the efficacious management of conjunctival hyperemia. The diagnostic techniques used for the grading of conjunctival hyperemia have also evolved from descriptive and subjective grading scales to more reliable computer-based objective grading scales (150).

In 1996, Owen et al. (151) developed a novel computer software method to quantify the conjunctival plexus on the scleral background to measure its vascular surface area from photographs. Huntjens et al. employed Advanced Ophthalmic Systems software, which uses an objective approach to grading conjunctival hyperemia, and found excellent repeatability and improved agreement between experienced and novice observers (152).

Research has begun to focus on the relationship between red eyes and dry eye, but the consistency between objective and subjective tests needs to be further strengthened. Schulze et al. quantified bulbar redness using the validated bulbar redness grading scale and an automated objective method (OCULUS Keratograph 5M) in participants with dry eye disease and nondry eye disease controls, but statistically significant differences in redness between the dry eye disease and control groups were only found using the validated bulbar redness scale (153).

### Other Examinations

#### Confocal Microscope

Confocal microscopy is a new technology that can aid in the *in vivo* assessment of structural changes in several ocular surface diseases on a cellular level. The application of IVCM in dry eye disease will be a powerful method to evaluate morphological changes in the ocular surface globally in the future (154). In the dry eye field, IVCM has been applied in the examination of the cornea, bulbar and palpebral conjunctiva, meibomian glands, and lacrimal glands (155–169).

First-generation confocal microscopy with an *in vivo* white-light through-focusing confocal microscope (Tandem Scanning Corp., Reston, VA) was developed and has advanced to an *in vivo* white-light slit-scanning confocal microscope (ConfoScan, Nidek Technologies, Vigonza, Veneto, Italy) (second generation). A new-generation *in vivo* laser-scanning confocal microscope (Heidelberg Retina Tomograph, Rostock Corneal Module; Heidelberg Engineering GmgH, Heidelberg, Baden-Württemberg, Germany) for corneal examination was recently reported to yield impressive, high-quality images in many corneal pathologies (169).

Confocal microscopy requires trained operators to acquire good quality scans but lacks built-in software to analyze nerves and inflammatory cells. As such, it is not used as often in clinical settings to diagnose dry eye. The relationship between corneal sensation and subbasal nerve morphology, as evaluated with IVCM, depends on the pathophysiological mechanism of ocular surface disease. In a recent review of the use of IVCM in dry eye, corneal subbasal nerves were implicated in the pathogenesis of the condition. There are many reports suggesting that corneal subbasal nerve density may decrease in dry eyes with Sjogren's syndrome (SS) (156, 158, 161). Ma et al. (165) found that a higher aggregated measure of tortuosity may be linked to ocular discomfort, visual function disturbance, and tear film instability. At the same time, Ma et al. (166) determined that objective visual quality is correlated with clinical symptoms and signs in patients with dry eye, which suggests that nerve changes may be a factor related to poor visual quality in these patients. Lin et al.'s study (167) showed that inflammatory dendritic cell density increases dramatically in the corneal epithelium of dry eye patients with non-SS and SS. Nerve fibers have an important influence on corneal tropism and contribute to the maintenance of healthy corneas. It has been postulated that inflammatory cells may induce a diminution of nerve fibers in the subbasal nerve plexus in dry eye patients with SS. The migration and maturation of dendritic cells are activated in response to proinflammatory stimulation. Dramatic increases in the number of dendritic cells in the corneal epithelium are thought to play a role in dry eye pathophysiology. Furthermore, some studies have determined that ocular Demodex mite infestation may be involved in ocular surface discomfort, inflammation, and meibomian gland dropout in patients with MGD (139, 168).

### Incomplete Blinking

Incomplete blinking is an important parameters of dry eye disease. Spontaneous blinking can be divided into complete blinking and incomplete blinking. Incomplete blinking was associated with a higher ocular surface disease index (OSDI) score, more meibomian gland dropout (MGD), and reduced tear film stability. LipiView II automatically detects and analyzes blink rate and blinking quality through the videos recorded. It displays the number of complete blinks and incomplete blinks and blink frequency numerically to help clinician to analyze blinking pattern (124).

## Outlook

The rapid progress in the development of dry eye diagnosis technologies has greatly promoted the level of diagnosis and treatment of dry eye and made dry eye a hot spot in the field of clinical ocular surfaces. In particular, the recent construction of a dry eye diagnosis and treatment center in China has received increased attention and developed rapidly (170). However, many areas worthy of exploration in the diagnosis of dry eye remained. These include the detection of the mechanism of dry eye-related neuropathy, *in vivo* functional examination of the meibomian glands, the correlation between tear film thickness, BUT, and noninvasive BUT, more accurate, and reliable tear and tear film examination methods. It is hoped that improvements in dry eye examination methods will promote all-round progress in the understanding, diagnosis, and treatment of dry eye and related researches (171).

## Data Availability Statement

The original contributions presented in the study are included in the article/supplementary material, further inquiries can be directed to the corresponding author/s.

## Author Contributions

XJ: conceptualization, writing—reviewing and editing, and supervision. YW: data curation, writing—original draft preparation, reviewing and editing, and visualization. CW: writing—original draft preparation. XW, KY, and YM: writing—original draft preparation. XH: writing—reviewing and editing. All authors contributed to the article and approved the submitted version.

## Funding

This work was supported by the National Natural Science Foundation of China: [Grant Numbers: 82171013 and 81870624] and Major Science and Technology Projects of Zhejiang Province [Grant Number: 2022C03173].

## Conflict of Interest

The authors declare that the research was conducted in the absence of any commercial or financial relationships that could be construed as a potential conflict of interest.

## Publisher's Note

All claims expressed in this article are solely those of the authors and do not necessarily represent those of their affiliated organizations, or those of the publisher, the editors and the reviewers. Any product that may be evaluated in this article, or claim that may be made by its manufacturer, is not guaranteed or endorsed by the publisher.
